# Development and validation of a national questionnaire assessing knowledge, attitudes, and practices on neglected tropical diseases among italian medical trainees

**DOI:** 10.1371/journal.pntd.0014644

**Published:** 2026-08-24

**Authors:** Giacomo Guido, Benedetta Rossi, Anna Barbiero, Claudia Mangiapelo, Federica Perego, Eleonora Perugini, Salvatore Scarso, Spinello Antinori, Alessandro Bartoloni, Nicoletta Basilico, Gioia Bongiorno, Fabrizio Bruschi, Susanna Capone, Antonio Cascio, Adriano Casulli, Serena Cavallero, Agnese Comelli, Angela Corpolongo, Marco Del Riccio, Giuseppina De Iaco, Marco Di Luca, Trentina Di Muccio, Giovanni Dolci, Alessandra D’Abramo, Stefano D’Amelio, Sara Epis, Guido Favia, Luisa Frallonardo, Flavia Riccardo, Beatrice Formenti, Simona Gabrielli, Samuele Gaggioli, Federico Gobbi, Roberta Iatta, Sergio Lo Caputo, Valentina Mangano, Francesca Mariotti, Rosalia Marrone, Alberto Matteelli, Caterina Monari, Emanuele Nicastri, Roberta Papagni, Mariantonietta Pisaturo, Marco Giuseppe Prato, Marco Pombi, Laura Rinaldi, Ilenia Annunziata Ritacco, Emanuele Rollo, Annalisa Saracino, Chiara Sepulcri, Francesca Serapide, Michele Spinicci, Natalia Tiberti, Carlo Torti, Lorenzo Zammarchi, Francesco Di Gennaro

**Affiliations:** 1 Department of Precision and Regenerative Medicine and Ionian Area (DiMePRe-J), University of Bari Aldo Moro, Bari, Italy; 2 Department of Clinical and Experimental Sciences, Unit of Infectious and Tropical Diseases, University of Brescia and ASST Spedali Civili di Brescia, Brescia, Italy; 3 Department of Experimental and Clinical Medicine, University of Florence, Florence, Italy; 4 Department of Infectious Diseases, Vector-Borne Diseases Unit, Istituto Superiore di Sanità, Rome, Italy; 5 Department of Public Health, Experimental and Forensic Medicine, University of Pavia, Pavia, Italy; 6 ParVirLab, Department of Biomedical, Surgical and Dental Sciences, University of Milan, Milan, Italy; 7 Department of Public Health and Infectious Diseases, Sapienza University of Rome, Rome, Italy; 8 Department of Infectious, Tropical Diseases and Microbiology, IRCCS Sacro Cuore Don Calabria Hospital, Negrar di Valpolicella, Verona, Italy; 9 Department of Biomedical and Clinical Sciences, Università degli Studi di Milano, Milano, Italy; 10 Department of Translational Research and New Technologies in Medicine and Surgery, University of Pisa, Pisa, Italy; 11 Department of Infectious and Tropical Diseases, University of Brescia and ASST Spedali Civili of Brescia, Brescia, Italy; 12 Department of Health Promotion, Mother and Child Care, Internal Medicine, and Medical Specialties “G D’Alessandro”, University of Palermo, Palermo, Italy; 13 European Union Reference Laboratory for Parasites (EURL-P). European Union Reference Laboratory for Public Health on Helminths and Protozoa (EURL-PH-HP). Unit of Foodborne and Neglected Parasitic Diseases. Department of Infectious Diseases, Istituto Superiore di Sanità, Rome, Italy; 14 Italian Society of Tropical Medicine and Global Health, Steering Committee, Viale Regina Elena 299, Rome, Italy; 15 Clinical and Research Department, National Institute for Infectious Diseases "Lazzaro Spallanzani" IRCCS, Rome, Italy; 16 Infectious Disease Unit, AUSL-IRCCS Reggio Emilia, Reggio Emilia, Italy; 17 EntoPar Lab, Department of Biosciences, University of Milan, Milan, Italy; 18 School of Bioscience and Veterinary Medicine, University of Camerino, Camerino, Italy; 19 Department of Infectious Diseases, Istituto Superiore di Sanità, Rome, Italy; 20 Fondazione Policlinico Gemelli IRCCS, Rome, Italy; 21 Department of Clinical and Experimental Sciences, University of Brescia, Brescia, Italy; 22 Interdisciplinary Department of Medicine, University of Bari, Bari, Italy; 23 Unit of Infectious Diseases, Department of Medical and Surgical Sciences, University of Foggia, Foggia, Italy; 24 Department of Translational Research and New Technologies in Medicine and Surgery, University of Pisa, Pisa, Italy; 25 National Institute for Health, Migration and Poverty (INMP), Via di San Gallicano 25/a, Rome, Italy; 26 Infectious Diseases Unit, Department of Mental Health and Public Medicine, University of Campania Luigi Vanvitelli, Naples, Italy; 27 USD Malattie Infettive e Tropicali, ASM Matera, Presidio Ospedaliero “Madonna delle Grazie”, Matera, Italy; 28 USL Toscana centro, Ospedale “San Jacopo”, Pistoia, Italy; 29 Department of Veterinary Medicine and Animal Production, CREMOPAR, University of Naples Federico II, Naples, Italy; 30 Department of Precision and Regenerative Medicine and Jonica Area, Department of Anesthesia and Intensive Care, University of Bari "Aldo Moro", Bari, Italy; 31 Department of Health Sciences, University of Genoa, Genova, Italy; 32 Department of Medical and Surgical Sciences, “Magna Graecia” University, Catanzaro, Italy; 33 Dipartimento di Sicurezza e Bioetica - Sezione di Malattie Infettive, Università Cattolica del Sacro Cuore, Rome, Italy; Italian Agency for Development Cooperation, SUDAN

## Abstract

**Background:**

Neglected Tropical Diseases (NTDs) affect nearly two billion people worldwide, yet preparedness to diagnose and manage them remains limited in high-income countries (HICs), where shifting epidemiology driven by climate changes, migration, mobility, international food and animal trade and global interconnection requires broader clinical competencies. Evidence on NTD-related knowledge among medical trainees in Europe is scarce. This study aimed to develop, validate, and field-test a comprehensive instrument assessing knowledge, attitudes, and practices (KAP) regarding NTDs among Italian medical students, recent graduates, and residents.

**Methods:**

A structured questionnaire was developed using a multi-step approach informed by World Health Organization (WHO) Neglected Tropical Diseases priorities and a targeted literature review. Content validity was assessed through a two-round Delphi process with a multidisciplinary national expert panel (Round 1: n = 36; Round 2: n = 34), followed by external expert evaluation (n = 17). Quantitative content validation included Item- and Scale-Level Content Validity Indices (I-CVI, S-CVI/Ave) and interquartile ranges (IQR) to assess expert consensus. Reliability and psychometric properties were evaluated in a national sample of Italian medical trainees (n = 96), including internal consistency (Cronbach’s α; KR-20), test–retest reliability (ICC; Cohen’s κ), and item performance analyses.

**Results:**

Across Delphi rounds, 91–98% of items reached consensus (IQR ≤ 1), and overall content validity was excellent (S-CVI/Ave 0.96–0.98). Reliability metrics confirmed strong internal consistency (α = 0.863; KR-20 = 0.82) and good temporal stability (ICC = 0.89; κ = 0.72–0.84). Known-groups validity was demonstrated by significantly higher knowledge scores among residents compared with medical students (p = 0.014).

**Conclusions:**

This study provides the first Delphi-validated national instrument assessing NTD-related knowledge among Italian medical trainees. The tool can offer a foundation for evaluating curricula and informing educational reform, supporting the integration of NTD-related competencies in European medical education in response to evolving global health challenges.

## Author summary

Neglected tropical diseases (NTDs) affect nearly 1.5 billion people worldwide and are increasingly becoming relevant beyond traditionally endemic regions due to climate change, migration, international travel, and globalization. Despite this, medical education in many high-income countries still provides limited training on these conditions. To address this gap, we developed and validated the first national questionnaire designed to assess knowledge, attitudes, and preparedness regarding NTDs among Italian medical students, recent graduates, and medical residents. The questionnaire was created through a rigorous multi-step process involving experts in infectious diseases, parasitology, public health, and medical education from across Italy. Validation included multiple Delphi rounds, external expert review, and psychometric testing among trainees. The final tool demonstrated excellent content validity, strong reliability, and the ability to distinguish different levels of expertise between students and residents. Our findings suggest that structured assessment tools can help identify educational gaps in tropical medicine and support curriculum improvements. As infectious diseases increasingly cross traditional geographic boundaries, strengthening awareness of NTDs among future physicians will be essential for timely diagnosis, equitable healthcare, and preparedness for emerging global health challenges.

## Introduction

The contemporary global health landscape is increasingly shaped by an intersecting set of crises: widening social and health inequities, accelerating environmental degradation, and growing human displacement driven by poverty, climate change, violence, and conflict [1–3]. Globalization, intensified human mobility, rapid urbanization, and global warming are altering the geographic distribution of diseases, reshaping the social determinants of health, and transforming patterns of disease transmission and access to care [4,5].

These forces place substantial pressure on health systems worldwide, which now face the dual challenge of rising burden of non-communicable diseases alongside the resurgence and geographic redistribution of infectious threats [5,6]. As a result, clinicians in all settings, not only in low- and middle-income countries (LMICs), must increasingly be equipped to recognize and manage conditions historically perceived as geographically distant or epidemiologically rare. This is particularly relevant within health systems striving to be migrant-sensitive and responsive to increasingly diverse population needs [3,7].

Neglected Tropical Diseases (NTDs) exemplify this shifting reality. Accounting for approximately 17% of global communicable disease burden, NTDs affect nearly 1.5 billion people, predominantly in LMICs [2,6]. The World Health Organization (WHO) currently recognizes twenty-one NTDs, many of which can lead to long-term disability, social stigma, and profound economic consequences when left untreated [8]. Despite their global relevance, awareness of NTDs remains limited among health professionals and policy makers in high-income countries, where the perceived burden is low and where educational curricula rarely include systematic training in tropical medicine [9]. This educational gap may contribute to delayed recognition, underdiagnosis, and limited preparedness among future physicians in high-income countries, where formal training in tropical medicine is often scarce or inconsistently integrated into medical curricula.

Yet epidemiological trends, including increased population mobility, vector redistribution linked to climate change, and the emergence of tropical infections in temperate regions, underscore the need for stronger preparedness [2,4]. To our knowledge, however, only a limited number of studies have evaluated awareness and knowledge of NTDs among medical students or early-career clinicians, and none have been conducted in Western Europe, to our knowledge [10]. This gap may reflect insufficient level of preparedness in high-income environments, where shifting epidemiological patterns require enhanced foundational training on NTDs [9–11]. Surveys from Africa, Asia, Latin America, and the Middle East consistently show significant knowledge gaps, limited diagnostic expertise, and inconsistent training [12, 13]. These findings indicate that comparable disparities in education may exist within European countries, such as Italy, which is facing growing exposure to the health consequences of climate change [14].

Therefore, this study aimed to develop and validate a comprehensive questionnaire to assess knowledge, attitudes, and practices regarding NTDs among Italian medical students, recent graduates, and residents. Preparedness within this group serves as proxy of overall health system readiness; therefore, the development and validation of a robust instrument can identify deficiencies, inform educational initiatives, and facilitate the integration of NTD competencies into core medical curricula.

## Methods

### Ethics Statement

This study was approved by the Ethics Committee of the Policlinico of Bari (Approval number 9881, dated 11 November 2025). All participants voluntarily participated in the study and provided written informed consent before questionnaire administration. Participation was anonymous, and all procedures were conducted in accordance with the Declaration of Helsinki and relevant national regulations.

### Study design and objectives

The study was conducted in a national multicenter academic setting involving Italian universities and affiliated teaching hospitals. The primary objective of this study was to develop and validate a structured questionnaire assessing awareness, knowledge, attitudes, and practices (KAP) regarding NTDs among Italian medical students, recent graduates, and medical residents. The tool was designed to evaluate the extent of formal training on NTDs, diagnostic preparedness, clinical experience, and interest in future education or research initiatives.

### Preparatory phase: Questionnaire development and first expert panel selection

**Fig 1:**
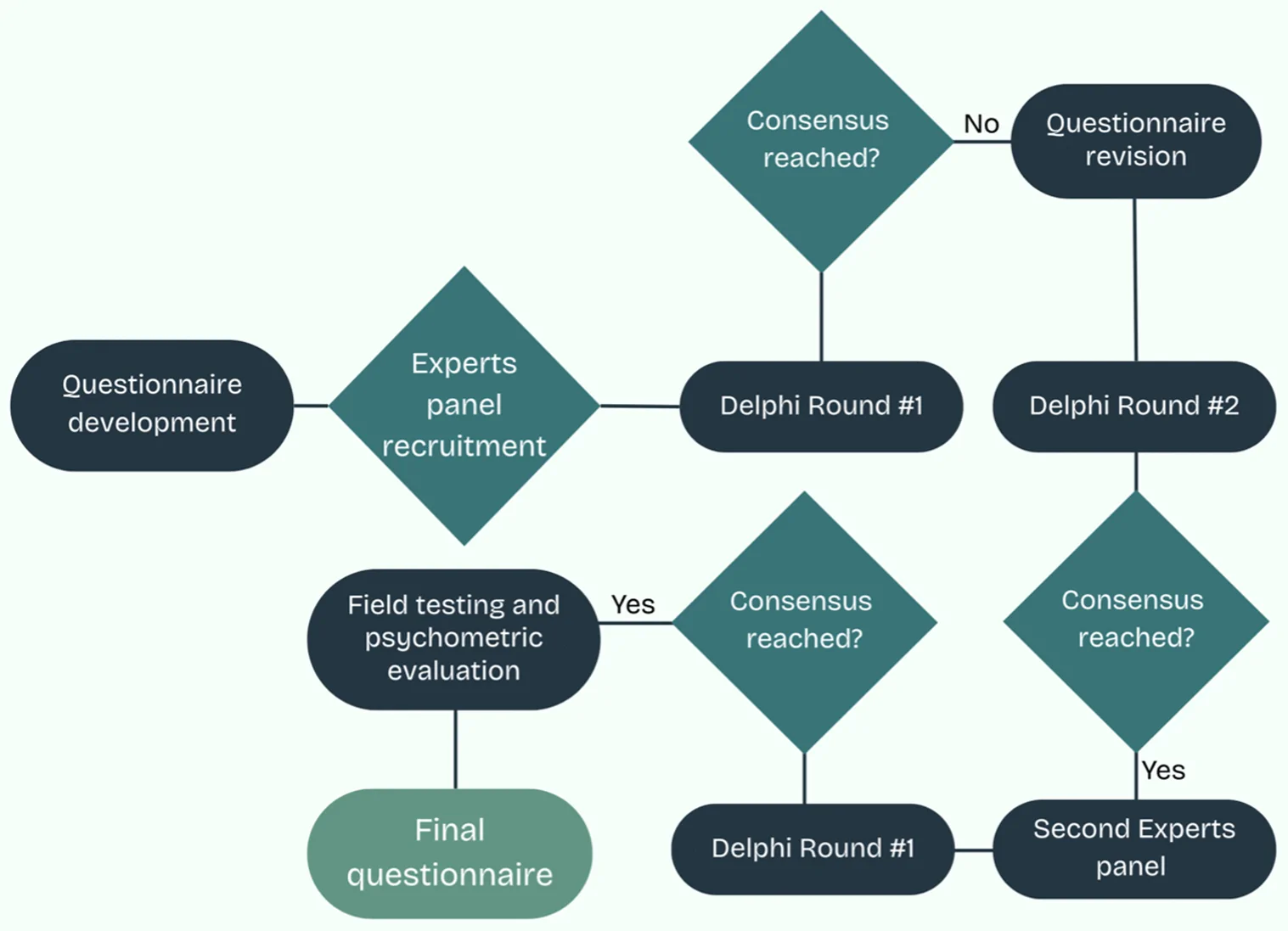
Flowchart of the questionnaire development and validation process.

The initial questionnaire was designed based on the WHO list of priority NTDs and current Italian university curricula in infectious and tropical diseases. The first version of the questionnaire was further informed by a non-systematic review of the literature of published studies and WHO reports, focusing on global priorities for NTD control, diagnostic challenges, and training gaps in medical education, until February 28th, 2025 (Fig 1). The survey comprised 43 questions divided into five sections:

- Demographic Data (age, gender, university, current position).
- Academic Training (exposure to NTD topics in curricula, perceived preparedness).
- Knowledge Assessment (clinical vignettes and diagnostic questions).
- Attitudes (perceptions of NTD relevance and One Health perspectives, Likert scale).
- Practice (experience with suspected cases, diagnostic requests, and extracurricular training).

Questions were designed as multiple-choice, 5-point Likert-scale, or dichotomous items. Pilot testing was conducted among a small sample (n ≈ 45) to refine clarity.

To ensure methodological rigour and content relevance, a multidisciplinary expert panel was assembled according to pre-defined criteria:

- Representation of national scientific societies: inclusion of experts from the Società Italiana di Parassitologia (SoIPa, www.soipa.it <http://www.soipa.it/>) and the Società Italiana di Medicina Tropicale e Salute Globale (SIMET, https://www.simetweb.eu <https://www.simetweb.eu/>), both members of the Italian Network for NTDs [15]
- Diversity of expertise: parasitologists, infectious disease specialists, clinicians, public health experts, and medical educators.
- Professional experience: at least half of the panel with ≥10 years of experience in tropical medicine, parasitology, or infectious disease education.
- Academic and policy perspective: participation of senior academics and contributors to national strategies on tropical and parasitic diseases.
- Early-career representation: at least two early-career researchers or residents actively engaged in NTD research or education.
- Student representation: inclusion of medical student representatives to ensure the questionnaire was relevant and accessible for its target audience.
- Gender balance.
- Geographical diversity: panelists drawn from medical schools and teaching hospitals across Northern, Central, and Southern Italy.

The final expert panel included 36 members, comprising representatives from SoIPa, SIMET, infectious disease specialists, parasitologists, medical educators, and student delegates, ensuring that the survey instrument was pedagogically robust, clinically accurate, and aligned with learner needs.

### Delphi survey

The Delphi method was used to gather expert opinions independently and reach consensus through iterative online surveys. Responses were anonymized, and only study coordinators accessed individual data, ensuring a double-blind process that minimized dominance bias. After each round, aggregated feedback was shared, enabling participants to revise their responses in light of group input. This structured and controlled approach promoted convergence of opinions while preserving individual autonomy.

Each question was evaluated using a 5-point Likert scale, with different assessment criteria based on question type:

- Questions from the Demographic Data were assessed for clarity, relevance, and appropriateness for the study objectives.
- Questions from Academic Training, Knowledge Assessment, Attitudes and Practices were evaluated for clarity, relevance, appropriateness, and ability to capture the internal dimensions of the study.

Participants were encouraged to provide qualitative feedback, including suggestions for additional indicators, refinements to survey items, and general comments to enhance the survey’s robustness.

### Content validity assessment

A single researcher conducted all statistical analyses to guarantee the validity of the development procedure. The content validity of the survey was assessed using two widely recognized indices: the Item Content Validity Index (I-CVI) and the Scale Content Validity Index (S-CVI/Ave) [16].

- I-CVI (Item Content Validity Index): The I-CVI was calculated for each item by dividing the number of experts who rated the item as “4” (relevant) or “5” (highly relevant) by the total number of experts. We pre-specified item-level thresholds to increase the conservativeness of content validation for a high-stakes instrument. In addition to the conventional cut-offs (I-CVI ≥ 0.78 acceptable; 0.70–0.78 revise; < 0.70 reject), we applied a more stringent banding (I-CVI ≥ 0.80 acceptable/revisions; 0.75–0.79 revise; < 0.74 reject). To ensure transparency, we report results under both schemes and provide modified kappa and 95% CIs for I-CVI.
- S-CVI/Ave (Scale Content Validity Index, Average Method): This index evaluates the overall validity of the survey by calculating the average I-CVI value across all items. An S-CVI/Ave ≥ 0.90 was considered indicative of strong content validity for the entire scale. Items with an S-CVI/Ave < 0.80 were flagged for major revision or removal, while those with an S-CVI/Ave between 0.80 and 0.89 were identified for minor revision.

The qualitative feedback supplemented the quantitative results from I-CVI and S-CVI/Ave calculations, enabling the formulation of data-informed decisions regarding the retention, revision, or exclusion of items. Items that were excluded from the final survey did not meet the I-CVI threshold in either of the Delphi sessions, despite the qualitative modifications.

To evaluate the degree of consensus among experts, the Interquartile Range (IQR) was computed for each item using the Likert scale ratings (1 –5) provided by the panel [16]. As a measure of variability in expert ratings, the IQR was calculated as the difference between the third quartile (Q3, 75th percentile) and the first quartile (Q1, 25th percentile):

- A low IQR (≤ (1) suggested that there was a high degree of agreement among experts, indicating a firm consensus on the item’s clarity, relevance, and appropriateness.
- Responses with a high IQR (> (1) exhibit a higher degree of variability, which may indicate a potential disagreement that necessitates additional revision or clarification in subsequent Delphi cycles.

By computing the IQR across expert ratings (1 = not pertinent to 5 = highly relevant), each survey item was analyzed. Items with an IQR between 0 and 1 were deemed to have achieved consensus, while those with an IQR > 1 were identified for further discussion and potential modification in the subsequent Delphi round. The IQR results were analyzed in conjunction with CVI measures to guarantee a thorough evaluation of content validity, thereby establishing a robust evaluation framework for the finalization and refinement of the survey.

### Second expert panel

To strengthen content validity, a second external panel of experts was recruited, composed of specialists not involved in the initial Delphi rounds. This panel included infectious disease clinicians, parasitologists, educators, and representatives from scientific societies, providing an independent review of the revised survey. The same evaluation criteria (I-CVI, S-CVI/Ave, IQR) were applied to confirm the clarity, relevance, and robustness of the questionnaire. This step minimized potential bias from repeated exposure among the first panel and enhanced the generalizability of the instrument.

### Reliability testing

A voluntary sample of 96 participants, including medical students, recent graduates, and residents from multiple Italian universities and specialties, was recruited to evaluate the psychometric reliability of the questionnaire. Participants were recruited through voluntary convenience sampling using institutional academic networks and professional contacts. Participants were selected to reflect the diversity of the survey’s target population, thereby enhancing representativeness and applicability across different stages of medical training.Given the overall sample n = 96 (and the smaller subgroup cell sizes), formal Differential Item Functioning (DIF) testing (Mantel-Haenszel, logistic DIF, or multi-group IRT) was not planned as a primary analysis due to limited power and unstable parameter estimates in small samples. Instead, we adopted a light DIF strategy to appraise item fairness across subgroups (students, residents, and recent graduates):

- Item performance by subgroup: inspection of item difficulty (p) and point-biserial discrimination separately within each subgroup.
- Reliability invariance: calculation of Cronbach’s α ± / KR-20 within subgroups to check consistency of internal reliability.
- Exploratory logistic checks (if flagged): for any item showing conspicuous subgroup discrepancies, we fitted binary logistic models (item score ~ total knowledge score + group) to probe potential bias, interpreting results cautiously and without formal multiplicity adjustments.

Together, these analyses confirmed that the instrument demonstrated high reproducibility, strong internal coherence, and robust reliability metrics, supporting its suitability for large-scale implementation in medical education and training contexts (S1 Data).

### Statistical analysis

Data were analysed using descriptive statistics (mean, standard deviation, and frequencies). Knowledge scores were computed as the percentage of correct responses. Content validity was assessed using the I-CVI, S-CVI/Ave, and IQR for consensus evaluation [17]. Reliability testing included test–retest analysis (Cohen’s kappa and intraclass correlation coefficients) and internal consistency assessment with Cronbach’s α for Likert-scale items and KR-20 for dichotomous items. All analyses were conducted in R (version 4.4.1, R Foundation for Statistical Computing, Vienna, Austria).

## Results

### Expert characteristics

The characteristics of the responders of both panels are reported in Table 1. Most participants (78%) had at least 10 years of experience at the time of the survey. All the inclusion criteria were met.

**Table 1:** Characteristics of the 53 experts composing both Panels.

	N (%)
**Sex**	
Male	25 (47)
Female	28 (53)
**Geographical Distribution**	
Northern Italy	14 (26)
Central Italy	26 (49)
Southern Italy	13 (25)
**Role**	
Academics	25 (47)
Clinicians	16 (30)
Young researchers	8 (15)
Student representatives	4 (8)

### Round 1 findings

The first Delphi round included 43 questions and was conducted from February 26, 2025, to March 20, 2025. Of the first invited expert panel, 28/36 (78%) completed the survey.

One reviewer analyzed the responses and assessed content validity using the S-CVI/Ave and I-CVI indices. Most questionnaire items demonstrated strong content validity and expert consensus during the first Delphi round. Four items with low content validity indices were removed after expert review because they were considered redundant, insufficiently informative, or potentially affected by recall bias. The questionnaire demonstrated excellent scale-level content validity across all evaluated domains (S-CVI/Ave range: 0.95–0.97), exceeding the conventional ≥0.90 benchmark. Detailed item-level modifications are reported in S1 Text.

The IQR analysis demonstrated a high level of consensus among experts: the mean IQR was 0.57 (median = 1.0, SD = 0.49; range: 0.0–1.25). Notably, 40.5% of items achieved an IQR of 0, indicating full agreement, while 97.6% of items had an IQR ≤ 1, confirming strong consensus across nearly all questions. Only 2.4% of items showed an IQR slightly above 1, suggesting minor variability in expert ratings.

The initial KAP questionnaire was also intended for nursing students; however, expert feedback indicated that the nursing curriculum in Italy focuses primarily on patient management rather than detailed disease-specific content. As a result, several knowledge-based items risked misrepresenting the actual scope of nursing education. To ensure the validity and fairness of the instrument, nursing students were subsequently excluded from the final survey sample.

### Round 2 findings

The second Delphi round included the selected 39 questions. Of the first invited expert panel, 29/36 completed the survey, resulting in an 81% response rate. The second round was conducted from April 17, 2025, to May 12, 2025.

One reviewer analysed the responses and assessed content validity using the S-CVI/Ave and I-CVI indices: most items demonstrated strong content validity during the second Delphi round and required only minor linguistic refinements. A small number of items underwent minor revisions, while one item with low relevance scores was removed following expert consensus because it was considered insufficiently reliable for assessing curricular exposure. Detailed item-level validity results are reported in the S1 Text.

The S-CVI/Ave in Round 2 is 0.96 for Clarity (C), 0.98 for Relevance (R), 0.98 for Appropriateness (A), and 0.97 for Ability to Capture the Intended Dimension. The overall S-CVI/Ave across dimensions is 0.9736, confirming excellent content validity.

The IQR analysis from this round demonstrated near-complete consensus among experts. The mean IQR was 0.05 (median = 0.00; SD = 0.22; range: 0.00–1.00). A total of 94.7% of items achieved an IQR of 0, indicating unanimous agreement, and 100% of items scored ≤1, confirming full consensus across all questionnaire items. No item exceeded an IQR of 1.

### Second panel findings

A second independent panel of experts was convened to validate the revised questionnaire developed through the initial two-round Delphi process. The panel consisted of other external 17 experts (11 women and 6 men), including 9 academics, 6 clinicians, 1 early-career researcher, and 1 student representative, ensuring multidisciplinary and learner-centred perspectives.

This second review confirmed the robustness of the revised tool, further enhancing its clarity, cultural relevance, and applicability to the target population. The second expert panel was conducted from June 30, 2025 to July 20, 2025.

The participation in the second expert panel was excellent, with 100% of invited experts submitting feedback. One reviewer analysed the responses and assessed content validity using the S-CVI/Ave and I-CVI indices: Most questionnaire items demonstrated strong content validity and required only minor linguistic refinements. A small number of items underwent minor wording revisions to improve clarity and consistency, including refinement of demographic terminology to avoid ambiguity. No items required removal during this phase, confirming the robustness of the questionnaire refinement process. Detailed item-level validity results are reported in the S1 Text.

The S-CVI/Ave for Clarity is 0.98, for Relevance 0.95, for Appropriateness 0.96, and for Ability to Capture the Intended Dimension 0.96; the overall S-CVI/Ave across dimensions is 0.96, indicating excellent content validity.

The IQR analysis further confirmed strong expert consensus. The mean IQR was 0.07 (median = 0.00; SD = 0.24; range: 0.00–1.00). A total of 91.9% of items achieved an IQR of 0, indicating unanimous agreement among experts, and 100% of items scored ≤1, confirming full consensus across all questions in this round.

The participation and feedback quality allowed the validation to be completed in a single round, given the high S-CVI scores already achieved in the initial Delphi phase.

### Reliability and validity

Following two iterative Delphi rounds and an external single-round validation confirming high content validity and consensus, the finalised questionnaire was field-tested in a sample of 96 participants (mean age 26.4 years, SD 3.2; 51 students, 34 residents: 12 surgical, 18 clinical, 4 services—and 11 recent graduates; 45% South, 21% Centre, 34% North) (Tables 2, 3). The study population included Italian medical students, recent graduates, and medical residents enrolled or trained at universities and teaching hospitals across Northern, Central, and Southern Italy. The analyses below report reliability, stability, item performance, known-groups, convergent validity, and fairness checks using the light DIF strategy.

**Table 2:** Structure of the questionnaire and content domains.

Section	Domain	Item content	Response format	N° of items
Section 1	Demographics	Age; sex at birth; university; current position; year of training; specialty	Open-ended / categorical	6
	Academic training	Exposure to NTDs during curricula; NTDs covered (WHO list); courses involved; perceived adequacy of training (diagnosis, treatment, prevention, public health); overall preparedness	Binary / multiple choice / Likert (1–5)	5
Section 2	Knowledge	Clinical vignettes (Dengue, Schistosomiasis, Geohelminths, Chagas disease, Echinococcosis, Cutaneous leishmaniasis); diagnostics; treatment; vectors; climate change impact	Multiple choice (single best answer)	15
Section 3	Attitudes	Importance of NTDs in curricula; public health relevance; interest in training; One Health perspective; research engagement	Likert scale (1–5)	5
Section 4	Practices	Previous suspicion/diagnosis of NTDs; participation in training activities; diagnostic suspicion based on country of origin; screening for schistosomiasis and *Strongyloides* spp	Binary / categorical	7

**Table 3:** Psychometric properties of analytical questionnaire domains.

Domain	Items (n)	Item type	Internal consistency	Test–retest reliability	Additional metrics
Knowledge	15	Dichotomous	KR-20 = 0.82	–	Item difficulty, discrimination
Attitudes	5	Likert (1–5)	Cronbach’s α = 0.863	ICC = 0.89	EFA, CFA
Practices	7	Binary	–	κ = 0.72–0.84	Agreement

### Internal consistency

- Attitudes (5 Likert items): Cronbach’s α = 0.863, indicating good internal consistency.
- Knowledge (15 dichotomous items, scored correct/incorrect): KR-20 = 0.82, indicating good reliability.

Temporal stability (test–retest)
A subsample of 40 participants repeated the survey after 2 weeks.
• Attitudes (composite of the 5 items): ICC (2,1) = 0.89; IC95% 0.83–0.93, showing good-to-excellent stability. SEM = 1.16; MDC95% = 3.22. The SEM of 1.16 reflects small absolute error, so individual changes ≥3.22 points (MDC95) can be considered real (beyond measurement error).
• Practice (7 binary items in the Practice section): Cohen’s κ across items ranged 0.72–0.84; a representative κ for an NTD-related screening item was 0.79, indicating substantial agreement

### Item analysis (knowledge)

All 15 knowledge items were profiled: item difficulty (p) ranged 0.42–0.78, and point-biserial discrimination ranged 0.28–0.52. No items met the pre-specified criteria for extreme difficulty (p < 0.20 or p > 0.90) or poor/negative discrimination; thus, no removals were warranted at this stage.

### Known-groups validity

Residents outperformed students on total knowledge (sum of 15 items): Welch’s t = 2.50, p = 0.014, Cohen’s d = 0.56 (a moderate effect), supporting known-groups validity.

### Construct validity

Exploratory factor analysis of the Attitudes items showed adequate sampling adequacy (KMO = 0.79; Bartlett’s test p < 0.001) and supported a unidimensional structure, explaining 58.6% of the variance, with all items showing salient loadings (>0.60). Confirmatory factor analysis confirmed good model fit (CFI = 0.96; TLI = 0.94; RMSEA = 0.06; SRMR = 0.04), with all standardized loadings statistically significant, supporting the construct validity of the Attitudes scale.

### Convergent validity

Self-reported preparedness correlated positively with total knowledge: Spearman’s ρ = 0.35, p < 0.001, consistent with moderate convergent validity.

### Fairness (light DIF)

Formal DIF testing was not performed given n = 96. Instead, we inspected item difficulty and discrimination by subgroup (students, residents, and recent graduates) for all 15 knowledge items, computed Cronbach’s α (attitudes, 5 items) and KR-20 (knowledge, 15 items) within subgroups and ran exploratory logistic checks for any flagged items. No systematic subgroup anomalies warranting item removal were identified.

The complete dataset and psychometric analyses are provided in S1 Data. The final validated questionnaire is provided in S1 Text.

## Discussion

This study presents the first validated national instrument designed to assess knowledge and preparedness regarding neglected tropical diseases among medical students and trainees in Italy. The development of the questionnaire through a multi-stage process, including a Delphi procedure, external expert validation, and psychometric testing, produced a tool capable of capturing multiple dimensions of NTD-related awareness.

Our research builds on previous studies conducted in Africa, Latin America, Asia, and the Middle East, which consistently report substantial gaps in knowledge of NTDs among medical students and young clinicians [12,18–22]. These findings highlight the importance of assessing whether similar educational gaps exist in Italy, a high-income country that is increasingly exposed to NTD-related risks due to climate changes, which may alter vector ecology, and growing human mobility, including movement of people who may carry asymptomatic NTDs [3,23].

The implications for medical education in Europe are substantial. Climate change, increased travel, migration, and global interconnectedness are reshaping the epidemiology of infectious diseases, rendering the traditional distinction between “tropical” and “non-tropical” illnesses increasingly artificial [24,25]. Medical curricula must therefore evolve accordingly. Integrating NTD-related skills into undergraduate and postgraduate training could strengthen clinical preparedness, improve diagnostic accuracy, and promote early recognition of uncommon presentations. The validated instrument may support curriculum mapping, evaluation of pre- and post-training educational interventions, inter-university comparisons, and assessment of targeted teaching modules in tropical medicine and global health. Moreover, enhanced awareness of NTDs may support the development of surveillance systems, research priorities, and intersectoral collaborations aligned with One Health principles.

For example, medical curricula often struggle to keep pace with the rapid evolution of global health challenges. Recent Italian data illustrate this gap: courses on climate change and health are rarely mandatory and are instead offered as elective modules, resulting in generally low levels of formal knowledge [26]. Interestingly, higher awareness has been documented among students reporting eco-anxiety, suggesting that concern for the future environmental and health threats may act as an informal driver of engagement with planetary health topics [26].

### Strenghts

A further strength of this study lies in the structure of the questionnaire, which combines factual items with short clinical cases. These case-based questions were included to assess not only theoretical knowledge but also the ability to recognise NTD-related presentations in practice. In addition, the Delphi process highlighted the value of involving students, early-career clinicians, and experts in the co-design and validation of educational tools, giving a meaningful role to those who are both current learners and future practitioners. The high level of consensus reached across diverse expert panels underscores the value of a participatory approach and ensures that the questionnaire reflects both pedagogical needs and clinical realities. Importantly, the composition of our panels was intentionally balanced, with representation of women and men, academic and non-academic professionals, and experts from Northern, Central, and Southern Italy, to minimise bias and ensure that the instrument incorporated a broad range of perspectives.

### Limitations

This study has some limitations. The relatively small sample size used for psychometric testing, particularly for confirmatory factor analysis and subgroup comparisons, may limit the robustness of some estimates. Therefore, these findings should be interpreted cautiously. In addition, the focus on Italian medical trainees may limit the generalisability of the results to other settings. In addition, the voluntary recruitment strategy may have introduced selection bias, and the self-reported nature of KAP surveys may be influenced by recall and social desirability biases. Finally, the limited sample size restricted the possibility of performing more advanced psychometric analyses, including formal differential item functioning testing. However, the methodological framework is transferable and could be applied to other countries seeking to assess gaps in tropical medicine education.

## Conclusions

In conclusion, knowledge of NTDs should no longer be confined to specialists; rather, it should form part of the foundational competencies of every future physician. This validated questionnaire provides a tool for assessing NTD-related competencies among future physicians in Italy. As global health challenges continue to evolve, integrating tropical medicine and NTD awareness into medical education will be essential to ensure preparedness, equity, and high-quality patient care across diverse epidemiological contexts. Future studies should evaluate the applicability of this instrument in larger and more diverse populations and explore its use for assessing the impact of educational interventions and curriculum reforms in tropical medicine and global health.

## Supporting information

S1 Data Raw dataset and results of item analysis, internal consistency, test–retest reliability, and construct validity analyses. (ZIP)

S1 Text Final validated questionnaire assessing knowledge, attitudes, and practices regarding neglected tropical diseases. (DOCX)

S1 Checklist CREDES checklist for the reporting of Delphi studies. (DOC)
