# Supplementary material for: Development and validation of a national questionnaire assessing knowledge, attitudes, and practices on neglected tropical diseases among italian medical trainees
Source: PLoS Negl Trop Dis. 2026 Aug 24;20(8):e0014644. doi: 10.1371/journal.pntd.0014644 (PMC13524332; doi:10.1371/journal.pntd.0014644)
Supplement: Supplementary file 1 [file pntd.0014644.s002.docx]

**S1 Appendix**

**Validated Questionnaire to Assess Knowledge, Attitudes, and Practices (KAP) on Neglected Tropical Diseases (NTDs) Among Medical Trainees**

**Section 1. Demographic Characteristics**

1. **Age:** ______
2. **Sex assigned at birth:**
   ☐ Female
   ☐ Male
   ☐ Prefer not to answer
3. **University currently attended / from which you graduated:**
4. **Current position:**
   ☐ Medical student
   ☐ Recent graduate
   ☐ Resident physician
5. **Year of training (if applicable):**
6. **Specialty (if applicable):**
   ☐ Internal Medicine
   ☐ Infectious and Tropical Diseases
   ☐ Pediatrics
   ☐ General Surgery
   ☐ Hygiene and Public Health
   ☐ Dermatology
   ☐ Gastroenterology
   ☐ Other (please specify): __________________

**Section 2. Academic Training**

1. During your academic training, were topics related to Neglected Tropical Diseases (NTDs) included in your curriculum?
   ☐ Yes
   ☐ No
   ☐ I do not know / I do not remember
2. If yes, which of the following NTDs were covered? (Multiple choice; based on WHO classification)

☐ Buruli ulcer
☐ American trypanosomiasis (Chagas disease)
☐ Dengue and Chikungunya
☐ Dracunculiasis (Guinea worm disease)
☐ Echinococcosis
☐ Foodborne trematodiases
☐ Human African trypanosomiasis
☐ Leishmaniasis
☐ Leprosy
☐ Lymphatic filariasis
☐ Mycetoma, chromoblastomycosis and other deep mycoses
☐ Noma
☐ Onchocerciasis
☐ Rabies
☐ Scabies and other ectoparasitoses
☐ Schistosomiasis
☐ Soil-transmitted helminthiases
☐ Snakebite envenoming
☐ Taeniasis / Neurocysticercosis
☐ Trachoma
☐ Yaws

3. In which course(s) were these topics primarily addressed? (Multiple choice)

☐ Infectious and Tropical Diseases
☐ Microbiology / Parasitology
☐ Laboratory Medicine
☐ Internal Medicine
☐ Urology
☐ Hygiene / Public Health
☐ Gynecology
☐ Dermatology
☐ Gastroenterology
☐ Elective courses
☐ Other (please specify): __________________
☐ These topics were not covered
☐ I do not know

4. Which aspects of NTDs were adequately addressed?
   (Response options: Yes / No / Partially / I do not know or do not remember)

- Diagnosis
- Therapeutic management
- Prevention and control
- Public health impact

5. To what extent did your academic training provide adequate preparation on NTDs?
   (1 = Very poor preparation; 5 = Excellent preparation)

Score: 1 ☐ 2 ☐ 3 ☐ 4 ☐ 5 ☐

**Section 3. Knowledge Assessment**

(Select one answer unless otherwise specified.)

1. Patient with high fever, retro-orbital headache, rash, gingival bleeding; recent travel to *Aedes aegypti* endemic area:
   ☐ Dengue
   ☐ Chikungunya
   ☐ Leishmaniasis
   ☐ Chagas disease
   ☐ I do not know
2. Sub-Saharan woman with hematuria and freshwater exposure:
   ☐ Schistosomiasis
   ☐ Echinococcosis
   ☐ Neurocysticercosis
   ☐ Leishmaniasis
   ☐ I do not know
3. Child from rural Niger with abdominal distension and diarrhea:
   ☐ Soil-transmitted helminthiasis
   ☐ Chagas disease
   ☐ Dengue
   ☐ Cystic echinococcosis
   ☐ I do not know
4. Bolivian patient with dilated cardiomyopathy:
   ☐ Chagas disease
   ☐ Visceral leishmaniasis
   ☐ Schistosomiasis
   ☐ Taeniasis / Neurocysticercosis
   ☐ I do not know
5. Romanian patient with hepatic cysts:
   ☐ Cystic echinococcosis
   ☐ Taeniasis / Neurocysticercosis
   ☐ Malaria
   ☐ Schistosomiasis
   ☐ I do not know
6. Afghan child with chronic painless ulcerative skin lesions:
   ☐ Cutaneous leishmaniasis
   ☐ Buruli ulcer
   ☐ Dengue
   ☐ Taeniasis / Neurocysticercosis
   ☐ I do not know
7. Most commonly used confirmatory test for Dengue:
   ☐ NS1 antigen rapid test or molecular testing
   ☐ Blood smear
   ☐ PCR for *Plasmodium*
   ☐ IgG ELISA
   ☐ I do not know
8. Main vector of Chikungunya virus:
   ☐ *Aedes aegypti*
   ☐ *Anopheles gambiae*
   ☐ *Culex pipiens*
   ☐ *Phlebotomus* spp.
   ☐ *Anopheles stephensi*
9. First-line treatment for *Ascaris lumbricoides*:
   ☐ Albendazole
   ☐ Ivermectin
   ☐ Doxycycline
   ☐ Amoxicillin
   ☐ Rifampicin
10. Reference diagnostic method for acute Chagas disease:
    ☐ Blood smear for *Trypanosoma cruzi*
    ☐ Blood culture
    ☐ Montenegro skin test
    ☐ Widal-Wright test
    ☐ Direct Coombs test
11. Gold standard for hepatic cystic echinococcosis diagnosis:
    ☐ Abdominal ultrasound
    ☐ Serology
    ☐ CT scan
    ☐ Whole-body MRI
    ☐ Stool examination
12. Most sensitive and specific test for visceral leishmaniasis:
    ☐ rK39-based serology
    ☐ Blood smear
    ☐ Chest X-ray
    ☐ Blood and stool cultures
    ☐ Antigen test
13. Mode of schistosomiasis transmission:
    ☐ Contact with freshwater contaminated by larvae
    ☐ Drinking contaminated water
    ☐ Insect bites
    ☐ Ingestion of infected meat
    ☐ Contact with infected individuals
14. Characteristic manifestation of neurocysticercosis:
    ☐ Recurrent seizures
    ☐ Jaundice
    ☐ Dyspepsia
    ☐ Persistent fever
    ☐ Meningitis
15. Effect of rising global temperatures on arboviral epidemiology:
    ☐ Increased cases in both tropical and temperate countries
    ☐ Increased in tropical and decreased in temperate countries
    ☐ No effect
    ☐ Decreased in tropical and increased in temperate countries
    ☐ Decreased in both

**Section 4. Attitudes**

(5-point Likert scale: 1 = Strongly disagree; 5 = Strongly agree)

- NTDs should be mandatory topics in healthcare curricula.
- NTDs are a priority public and global health issue, including in high-income countries.
- I am willing to attend extracurricular NTD training courses.
- I would participate in workshops addressing NTDs and One Health interconnections.
- I am interested in collaborating on NTD-related research or prevention projects.

**Section 5. Practices**

1. Have you ever diagnosed or suspected a case of NTD?
   ☐ Yes
   ☐ No
   ☐ I do not remember
   ☐ Not applicable
2. Have you participated in NTD-related seminars, webinars, conferences, or elective activities?
   ☐ Yes
   ☐ No
   ☐ I do not remember

If yes, main motivation:
☐ Personal initiative
☐ Mandatory academic training
☐ NGO or student association activity
☐ Other: __________________

3. Have you ever requested diagnostic testing for NTDs based on a patient’s country of origin?
   ☐ Yes
   ☐ No
   ☐ I do not know / I do not remember
   ☐ Not applicable
4. Have you ever requested screening for schistosomiasis?
   ☐ Yes
   ☐ No
   ☐ I do not know / I do not remember
   ☐ Not applicable
5. Have you ever requested screening for *Strongyloides* spp.?
   ☐ Yes
   ☐ No
   ☐ I do not know / I do not remember
   ☐ Not applicable
