# Supplementary material for: Development and validation of a national questionnaire assessing knowledge, attitudes, and practices on neglected tropical diseases among italian medical trainees
Source: PLoS Negl Trop Dis. 2026 Aug 24;20(8):e0014644. doi: 10.1371/journal.pntd.0014644 (PMC13524332; doi:10.1371/journal.pntd.0014644)
Supplement: Supplementary file 2 [file pntd.0014644.s004.docx]

**Journal Requirements:**

1) Please ensure that the CRediT author contributions listed for every co-author are completed accurately and in full.

At this stage, the following Authors/Authors require contributions: Anna Barbiero, Claudia Mangiapelo, Federica Perego, Eleonora Perugini, Salvatore Scarso, Spinello Antinori, Alessandro Bartoloni, Nicoletta Basilico, Gioia Bongiorno, Fabrizio Bruschi, Susanna Capone, Antonio Cascio, Adriano Casulli, Serena Cavallero, Agnese Comelli, Angela Corpolongo, Marco Del Riccio, Giuseppina De Iaco, Marco Di Luca, Trentina Di Muccio, Giovanni Dolci, Alessandra D’Abramo, Stefano D’Amelio, Sara Epis, Guido Favia, Luisa Frallonardo, Flavia Riccardo, Beatrice Formenti, Simona Gabrielli, Samuele Gaggioli, Federico Gobbi, Roberta Iatta, Sergio Lo Caputo, Valentina Mangano, Francesca Mariotti, Rosalia Marrone, Alberto Matteelli, Caterina Monari, Emanuele Nicastri, Roberta Papagni, Mariantonietta Pisaturo, Marco Giuseppe Prato, Marco Pombi, Laura Rinaldi, Ilenia Annunziata Ritacco, Emanuele Rollo, Annalisa Saracino, Chiara Sepulcri, Francesca Serapide, Michele Spinicci, Natalia Tiberti, Carlo Torti, and Lorenzo Zammarchi. Please ensure that the full contributions of each author are acknowledged in the "Add/Edit/Remove Authors" section of our submission form.
The list of CRediT author contributions may be found here: <https://journals.plos.org/plosntds/s/authorship#loc-author-contributions>

**A:** Thanks for your comment, we updated the list in the proper section.

2) Please provide an Author Summary. This should appear in your manuscript between the Abstract (if applicable) and the Introduction, and should be 150-200 words long. The aim should be to make your findings accessible to a wide audience that includes both scientists and non-scientists. Sample summaries can be found on our website under Submission Guidelines:
   <https://journals.plos.org/plosntds/s/submission-guidelines#loc-parts-of-a-submission>

**A:** Thanks, we uploaded this section between the Abstract and the Introduction as suggested.

3) Please insert an Ethics Statement at the beginning of your Methods section, under a subheading 'Ethics Statement'. It must include:

i) The full name(s) of the Institutional Review Board(s) or Ethics Committee(s)
ii) The approval number(s), or a statement that approval was granted by the named board(s)
iii) A statement that formal consent was obtained (must state whether verbal/written) OR the reason consent was not obtained (e.g. anonymity). NOTE: If child participants, the statement must declare that formal consent was obtained from the parent/guardian.].

**A:** We thank the Editors for this important suggestion. An “Ethics Statement” subsection has now been added at the beginning of the Methods section, including the name of the Ethics Committee, approval details, and information regarding informed consent obtained from participants prior to participation.

4) Please upload all main figures as separate Figure files in .tif or .eps format. For more information about how to convert and format your figure files please see our guidelines:
   <https://journals.plos.org/plosntds/s/figures>

**A:** Thanks for your comment, we uploaded Figure file in .tiff.

5) We have noticed that you have uploaded Supporting Information files, but you have not included a list of legends. Please add a full list of legends for your Supporting Information files after the references list.

**A:** Corrected in the Methods section as suggested.

6) We note that your Data Availability Statement is currently as follows: "All relevant data supporting the findings of this study are included within the manuscript and its Supporting Information files.". Please confirm at this time whether or not your submission contains all raw data required to replicate the results of your study. Authors must share the “minimal data set” for their submission. PLOS defines the minimal data set to consist of the data required to replicate all study findings reported in the article, as well as related metadata and methods (<https://journals.plos.org/plosone/s/data-availability#loc-minimal-data-set-definition>).
   For example, authors should submit the following data: 
   1) The values behind the means, standard deviations and other measures reported;
   2) The values used to build graphs;
   3) The points extracted from images for analysis..
   Authors do not need to submit their entire data set if only a portion of the data was used in the reported study.
   If your submission does not contain these data, please either upload them as Supporting Information files or deposit them to a stable, public repository and provide us with the relevant URLs, DOIs, or accession numbers. For a list of recommended repositories, please see <https://journals.plos.org/plosone/s/recommended-repositories>. 
   If there are ethical or legal restrictions on sharing a de-identified data set, please explain them in detail (e.g., data contain potentially sensitive information, data are owned by a third-party organization, etc.) and who has imposed them (e.g., an ethics committee). Please also provide contact information for a data access committee, ethics committee, or other institutional body to which data requests may be sent. If data are owned by a third party, please indicate how others may request data access.

**A:** Thanks, we apologise for not uploading the supporting data. We updated with the correct supporting file containing all information for the statistical analysis

Your current Financial Disclosure states, "The author(s) received no specific funding for this work.".
However, your funding information on the submission form indicates receiving funds. Please ensure that the funders and grant numbers match between the Financial Disclosure field and the Funding Information tab in your submission form. Note that the funders must be provided in the same order in both places as well.Please amend your detailed Financial Disclosure statement. This is published with the article. It must therefore be completed in full sentences and contain the exact wording you wish to be published.
1) Please clarify all sources of financial support for your study. List the grants, grant numbers, and organizations that funded your study, including funding received from your institution. Please note that suppliers of material support, including research materials, should be recognized in the Acknowledgements section rather than in the Financial Disclosure
2) State the initials, alongside each funding source, of each author to receive each grant. For example: "This work was supported by the National Institutes of Health (####### to AM; ###### to CJ) and the National Science Foundation (###### to AM)."
3) State what role the funders took in the study. If the funders had no role in your study, please state: "The funders had no role in study design, data collection and analysis, decision to publish, or preparation of the manuscript."
4) If any authors received a salary from any of your funders, please state which authors and which funders..

**A:** This study did not receive specific funding. Some authors receive institutional or research support from the Italian Ministry of Health (“Fondi Ricerca Corrente”) to their respective institutions; however, these funds did not directly support the design, conduct, analysis, or publication of the present study. We updated the Financial Disclosure statement as you suggested.

**Reviewers' Comments:**

Reviewer's Responses to Questions

**Key Review Criteria Required for Acceptance?**

As you describe the new analyses required for acceptance, please consider the following:

**Methods**

-Are the objectives of the study clearly articulated with a clear testable hypothesis stated?

-Is the study design appropriate to address the stated objectives?

-Is the population clearly described and appropriate for the hypothesis being tested?

-Is the sample size sufficient to ensure adequate power to address the hypothesis being tested?

-Were correct statistical analysis used to support conclusions?

-Are there concerns about ethical or regulatory requirements being met?

Reviewer #1: Are the objectives of the study clearly articulated with a clear testable hypothesis stated?
Yes. The objectives are clearly articulated and the study addresses a well-defined and testable research question.
Is the study design appropriate to address the stated objectives?
Yes. The study design is appropriate and adequately aligned with the stated objectives.
Is the population clearly described and appropriate for the hypothesis being tested?
Yes. The study population is clearly described and appropriate for the research question.
Is the sample size sufficient to ensure adequate power to address the hypothesis being tested?
Yes. The sample size appears adequate to support the analyses and the study objectives.
Were correct statistical analyses used to support conclusions?
Yes. The statistical analyses are appropriate and support the conclusions drawn.
Are there concerns about ethical or regulatory requirements being met?
No. There are no major concerns regarding ethical or regulatory requirements based on the information provided.

**A:** We sincerely thank the Reviewer for the positive and constructive evaluation of our manuscript. We appreciate the acknowledgment of the clarity of the study objectives, the appropriateness of the study design and statistical analyses, and the overall methodological rigor of the work. We are also grateful for the positive assessment regarding the study population, sample size, and ethical aspects of the study.

Reviewer #2: The method was clear,well prepared and detailed however minor revision required, please add and clearly state the study population, setting and the sampling in their specific appropriate sections.

**A:** We thank the Reviewer for this helpful suggestion. We have revised the Methods section to more clearly describe the study population, study setting, and sampling approach in their respective sections. In particular, we clarified the characteristics of the included participants, the multicentre national academic setting, and the voluntary recruitment strategy adopted for both the Delphi validation process and the psychometric testing phase.

Reviewer #3: -Clarify recruitment strategy and representativeness
- Explicit statement of: Ethics approval (with reference number), Informed consent
-The sample size used for psychometric validation is relatively small, especially for analyses such as CFA and subgroup comparisons. While acceptable for an initial validation study, this limitation should be more explicitly acknowledged, and conclusions should be framed accordingly to avoid overgeneralization.

**A:** We thank the Reviewer for these important observations. We have now added an explicit “Ethics Statement” subsection at the beginning of the Methods section, including the Ethics Committee approval details and information regarding written informed consent obtained from participants. In addition, we expanded the limitations section to more clearly acknowledge the relatively small sample size used for psychometric validation, particularly for confirmatory factor analysis and subgroup comparisons. The conclusions were also slightly moderated to avoid overgeneralization and to emphasize the preliminary nature of this national validation study.

**Results**

-Does the analysis presented match the analysis plan?

-Are the results clearly and completely presented?

-Are the figures (Tables, Images) of sufficient quality for clarity?

Reviewer #1: Does the analysis presented match the analysis plan?
Yes. The analyses presented are consistent with the study aims and appear to match the analysis plan.
Are the results clearly and completely presented?
Yes. The results are clearly organized and sufficiently presented.
Are the figures (Tables, Images) of sufficient quality for clarity?
Yes. The tables and figures are clear, appropriate, and of sufficient quality to support the manuscript.

**A:** We sincerely thank the Reviewer for the positive evaluation of the manuscript.

Reviewer #2: The results were organized and reported the findings of the study objectives; however, no items were cited or mentioned and no scale attached.

**A:** We thank the Reviewer for this helpful observation. Although mistakenly not cited in the manuscript, the questionnaire was added as Supporting File. We appropriately cited it within the manuscript to improve transparency and reproducibility.

Reviewer #3: - Currently, it is dense with numerical detail, which may hinder readability. Streamlining the presentation and moving highly detailed outputs to supplementary files would improve accessibility.

**A:** We thank the Reviewer for this constructive suggestion. To improve readability and accessibility, we streamlined parts of the Results section by reducing excessive numerical detail in the main text and relocating selected detailed outputs to the Supplementary Results S1.

**Conclusions**

-Are the conclusions supported by the data presented?

-Are the limitations of analysis clearly described?

-Do the authors discuss how these data can be helpful to advance our understanding of the topic under study?

-Is public health relevance addressed?

Reviewer #1: Are the conclusions supported by the data presented?
Yes. The conclusions are supported by the data presented and remain appropriately balanced.
Are the limitations of analysis clearly described?
Yes. The main limitations are acknowledged and discussed adequately.
Do the authors discuss how these data can be helpful to advance our understanding of the topic under study?
Yes. The manuscript clearly explains how these findings contribute to advancing knowledge in the field.
Is public health relevance addressed?
Yes. The public health relevance is clearly addressed and represents one of the strengths of the manuscript.

**A:** We sincerely thank the Reviewer for the positive evaluation of the manuscript and for acknowledging the balance of the conclusions, the discussion of limitations, and the public health relevance of the study.

Reviewer #2: The conclusion supported with the data but it require revision and clarity and it should be separated from the discussion and kindly organized it with future interventions which help advance the topic and the outcomes of this study.
Please provide a section for the strength and limitations of this study as were not stated in this version of manuscript.

**A:** We thank the Reviewer for these constructive suggestions. We revised and clarified the Conclusions section, separating it more clearly from the Discussion and expanding the perspectives on future educational and research interventions that may build on the findings of this study. In addition, the main strengths and limitations of the study are now discussed as separated sub-sections of the Discussion

Reviewer #3: - the limitations section should be expanded to include not only sample size constraints but also potential selection bias, limitations inherent to KAP surveys, and constraints in psychometric testing (e.g., limited power for DIF analysis).

**A:** We thank the Reviewer for this important observation. The limitations section has now been expanded to include not only the relatively small sample size used for psychometric validation, but also the potential for selection bias related to voluntary participation, limitations inherent to KAP-based survey studies, and the limited statistical power available for advanced psychometric analyses such as formal DIF testing.

**Editorial and Data Presentation Modifications?**

Use this section for editorial suggestions as well as relatively minor modifications of existing data that would enhance clarity. If the only modifications needed are minor and/or editorial, you may wish to recommend “Minor Revision” or “Accept”.

Reviewer #1: Minor revision

**A:** We thank the Reviewer for the positive evaluations of our manuscript.

Reviewer #2: For the title please revise it to be more reflective and interesting, add the population and revise it.

The heading abstract word is missed, and at the conclusion of the abstract stated that,the study aims to assess the knowledge without mentioning the other objectives. Please add the attitude and practice and rephrase the conclusion to be well reflected.

In the justification of the background please add how this study addresses a gap and identifies how it provides an unexisting instrument.

**A:** We thank the Reviewer for these helpful suggestions. We revised the title to better reflect the study population and scope of the work. We also corrected the abstract formatting, added explicit reference to knowledge, attitudes, and practices (KAP) in the abstract conclusions, and revised the wording for improved clarity and consistency with the study objectives. In addition, we strengthened the background section to more clearly highlight the existing gap in the literature and the novelty of developing a validated national instrument for assessing NTD-related preparedness among medical trainees.

Reviewer #3: (No Response)

**Summary and General Comments**

Use this section to provide overall comments, discuss strengths/weaknesses of the study, novelty, significance, general execution and scholarship. You may also include additional comments for the author, including concerns about dual publication, research ethics, or publication ethics. If requesting major revision, please articulate the new experiments that are needed.

Reviewer #1: This is a timely, relevant, and well-written manuscript addressing an important educational and public health gap. The topic is highly appropriate for the journal, and the authors should be commended for focusing on preparedness regarding neglected tropical diseases in a high-income European context, where these competencies are often underestimated despite changing epidemiological patterns. Overall, I believe the manuscript is suitable for publication after minor revisions, mainly aimed at improving the clarity, conciseness, and interpretative strength. Below my suggestions:

1.The rationale is strong and convincing, but the final paragraph currently repeats the study aim almost verbatim. I suggest removing the duplicated sentence and slightly tightening the transition from the broader global-health framing to the specific Italian educational context. This would improve readability and make the study objective stand out more clearly.

2.Please improve the balance of the Introduction by focusing more directly on the educational gap.
The opening of the Introduction is broad and well contextualized within globalization, migration, climate change, and health inequities, which is valuable. However, the educational dimension of the problem emerges somewhat late. I would recommend bringing forward, a little earlier, the point that the core issue is insufficient preparedness among medical trainees in high-income settings, so that the narrative becomes more immediately aligned with the specific purpose of the paper.


3. The Discussion appropriately argues that preparedness among young physicians may reflect broader health-system readiness. However, at present these two levels are somewhat blended. I suggest separating more clearly what the study directly supports—namely, curriculum assessment and educational gap identification—from the broader downstream implications for surveillance, preparedness, and health-system responsiveness. This would make the interpretation more precise and proportionate to the study findings.

4, Please expand the practical implications of the tool for curriculum development.
The Discussion rightly emphasizes that the instrument may inform educational reform, but this point could be made even more useful by adding a few concrete examples of application. For instance, the authors might briefly mention its potential use for curriculum mapping, pre/post educational interventions, inter-university comparisons, or assessment of targeted teaching modules in tropical medicine or global health. This would increase the practical value of the paper for educators and policy makers.
5.
The final message is compelling, but it could be strengthened by outlining what the next logical step should be after validation of this national tool. For example, the authors could mention future multicenter use across Italian universities, periodic reassessment of preparedness over time, or eventual adaptation of the instrument for broader European contexts. This would give the Discussion a stronger forward-looking perspective and highlight the manuscript’s wider relevance.

**A:** We sincerely thank the Reviewer for the constructive and positive evaluation of our manuscript.

- We revised the final paragraph of the Introduction to remove redundancy and improve the transition between the broader global health context and the specific educational gap addressed in Italy.
- We strengthened the educational perspective earlier in the Introduction to better emphasize the limited preparedness regarding NTDs among medical trainees in high-income settings.
- We revised the Discussion to more clearly distinguish the direct educational implications of the study from the broader implications for health-system preparedness.
- We expanded the practical implications of the validated instrument by including examples of potential applications in curriculum assessment and educational interventions.
- Finally, we strengthened the future perspectives of the manuscript by discussing possible multicentre implementation and future adaptation of the tool in broader European settings

Reviewer #2: This study was well structured, sequenced and organized, however some revisions required specifically in the title of this study, the study justification and conclusion.

**A:** We thank the Reviewer for the valuable suggestions and the positive evaluations of the manuscript.

Reviewer #3: (No Response)
