# Supplementary material for: Development and validation of a national questionnaire assessing knowledge, attitudes, and practices on neglected tropical diseases among italian medical trainees
Source: PLoS Negl Trop Dis. 2026 Aug 24;20(8):e0014644. doi: 10.1371/journal.pntd.0014644 (PMC13524332; doi:10.1371/journal.pntd.0014644)
Supplement: Peer review history

# Peer review: Decision Letter 0

**DOI:** 10.1371/journal.pntd.0014644.r001

**Authors:** Sitara Swarna Rao Ajjampur, Damiano Pizzol

8 May 2026

PNTD-D-26-00401

Validated National Tool to Assess Preparedness of Young Physicians on Neglected Tropical Diseases

PLOS Neglected Tropical Diseases

Dear Dr. GUIDO,

Thank you for submitting your manuscript to PLOS Neglected Tropical Diseases. After careful consideration, we feel that it has merit but does not fully meet PLOS Neglected Tropical Diseases's publication criteria as it currently stands. Therefore, we invite you to submit a revised version of the manuscript that addresses the points raised during the review process.

Please submit your revised manuscript within by Jul 07 2026 11:59PM. If you will need more time than this to complete your revisions, please reply to this message or contact the journal office at plosntds@plos.org. When you're ready to submit your revision, log on to https://www.editorialmanager.com/pntd/ and select the 'Submissions Needing Revision' folder to locate your manuscript file.

Please include the following items when submitting your revised manuscript:

* A letter that responds to each point raised by the editor and reviewer(s). You should upload this letter as a separate file labeled 'Response to Reviewers'. This file does not need to include responses to any formatting updates and technical items listed in the 'Journal Requirements' section below.

* A marked-up copy of your manuscript that highlights changes made to the original version. You should upload this as a separate file labeled 'Revised Manuscript with Track Changes'.

* An unmarked version of your revised paper without tracked changes. You should upload this as a separate file labeled 'Manuscript'.

If you would like to make changes to your financial disclosure, competing interests statement, or data availability statement, please make these updates within the submission form at the time of resubmission. Guidelines for resubmitting your figure files are available below the reviewer comments at the end of this letter.

We look forward to receiving your revised manuscript.

Kind regards,

Damiano Pizzol

Guest Editor

PLOS Neglected Tropical Diseases

Sitara Ajjampur

Section Editor

PLOS Neglected Tropical Diseases

Shaden Kamhawi

co-Editor-in-Chief

PLOS Neglected Tropical Diseases

orcid.org/0000-0003-4304-636XX

Paul Brindley

co-Editor-in-Chief

PLOS Neglected Tropical Diseases

orcid.org/0000-0003-1765-0002

**Journal Requirements:**

1) Please ensure that the CRediT author contributions listed for every co-author are completed accurately and in full.

At this stage, the following Authors/Authors require contributions: Anna Barbiero, Claudia Mangiapelo, Federica Perego, Eleonora Perugini, Salvatore Scarso, Spinello Antinori, Alessandro Bartoloni, Nicoletta Basilico, Gioia Bongiorno, Fabrizio Bruschi, Susanna Capone, Antonio Cascio, Adriano Casulli, Serena Cavallero, Agnese Comelli, Angela Corpolongo, Marco Del Riccio, Giuseppina De Iaco, Marco Di Luca, Trentina Di Muccio, Giovanni Dolci, Alessandra D’Abramo, Stefano D’Amelio, Sara Epis, Guido Favia, Luisa Frallonardo, Flavia Riccardo, Beatrice Formenti, Simona Gabrielli, Samuele Gaggioli, Federico Gobbi, Roberta Iatta, Sergio Lo Caputo, Valentina Mangano, Francesca Mariotti, Rosalia Marrone, Alberto Matteelli, Caterina Monari, Emanuele Nicastri, Roberta Papagni, Mariantonietta Pisaturo, Marco Giuseppe Prato, Marco Pombi, Laura Rinaldi, Ilenia Annunziata Ritacco, Emanuele Rollo, Annalisa Saracino, Chiara Sepulcri, Francesca Serapide, Michele Spinicci, Natalia Tiberti, Carlo Torti, and Lorenzo Zammarchi. Please ensure that the full contributions of each author are acknowledged in the "Add/Edit/Remove Authors" section of our submission form.

The list of CRediT author contributions may be found here: https://journals.plos.org/plosntds/s/authorship#loc-author-contributions

2) Please provide an Author Summary. This should appear in your manuscript between the Abstract (if applicable) and the Introduction, and should be 150-200 words long. The aim should be to make your findings accessible to a wide audience that includes both scientists and non-scientists. Sample summaries can be found on our website under Submission Guidelines:

https://journals.plos.org/plosntds/s/submission-guidelines#loc-parts-of-a-submission

3) Please insert an Ethics Statement at the beginning of your Methods section, under a subheading 'Ethics Statement'. It must include:

i) The full name(s) of the Institutional Review Board(s) or Ethics Committee(s)

ii) The approval number(s), or a statement that approval was granted by the named board(s)

iii) A statement that formal consent was obtained (must state whether verbal/written) OR the reason consent was not obtained (e.g. anonymity). NOTE: If child participants, the statement must declare that formal consent was obtained from the parent/guardian.].

4) Please upload all main figures as separate Figure files in .tif or .eps format. For more information about how to convert and format your figure files please see our guidelines:

https://journals.plos.org/plosntds/s/figures

5) We have noticed that you have uploaded Supporting Information files, but you have not included a list of legends. Please add a full list of legends for your Supporting Information files after the references list.

6) We note that your Data Availability Statement is currently as follows: "All relevant data supporting the findings of this study are included within the manuscript and its Supporting Information files.". Please confirm at this time whether or not your submission contains all raw data required to replicate the results of your study. Authors must share the “minimal data set” for their submission. PLOS defines the minimal data set to consist of the data required to replicate all study findings reported in the article, as well as related metadata and methods (https://journals.plos.org/plosone/s/data-availability#loc-minimal-data-set-definition).

For example, authors should submit the following data:

1) The values behind the means, standard deviations and other measures reported;

2) The values used to build graphs;

3) The points extracted from images for analysis..

Authors do not need to submit their entire data set if only a portion of the data was used in the reported study.

If your submission does not contain these data, please either upload them as Supporting Information files or deposit them to a stable, public repository and provide us with the relevant URLs, DOIs, or accession numbers. For a list of recommended repositories, please see https://journals.plos.org/plosone/s/recommended-repositories.

If there are ethical or legal restrictions on sharing a de-identified data set, please explain them in detail (e.g., data contain potentially sensitive information, data are owned by a third-party organization, etc.) and who has imposed them (e.g., an ethics committee). Please also provide contact information for a data access committee, ethics committee, or other institutional body to which data requests may be sent. If data are owned by a third party, please indicate how others may request data access.

7) Your current Financial Disclosure states, "The author(s) received no specific funding for this work.".

However, your funding information on the submission form indicates receiving funds. Please ensure that the funders and grant numbers match between the Financial Disclosure field and the Funding Information tab in your submission form. Note that the funders must be provided in the same order in both places as well.

Please amend your detailed Financial Disclosure statement. This is published with the article. It must therefore be completed in full sentences and contain the exact wording you wish to be published.

1) Please clarify all sources of financial support for your study. List the grants, grant numbers, and organizations that funded your study, including funding received from your institution. Please note that suppliers of material support, including research materials, should be recognized in the Acknowledgements section rather than in the Financial Disclosure

2) State the initials, alongside each funding source, of each author to receive each grant. For example: "This work was supported by the National Institutes of Health (####### to AM; ###### to CJ) and the National Science Foundation (###### to AM)."

3) State what role the funders took in the study. If the funders had no role in your study, please state: "The funders had no role in study design, data collection and analysis, decision to publish, or preparation of the manuscript."

4) If any authors received a salary from any of your funders, please state which authors and which funders..

Note: If the reviewer comments include a recommendation to cite specific previously published works, please review and evaluate these publications to determine whether they are relevant and should be cited. There is no requirement to cite these works unless the editor has indicated otherwise.

**Reviewers' Comments:**

Reviewer's Responses to Questions

**Key Review Criteria Required for Acceptance?**

As you describe the new analyses required for acceptance, please consider the following:

**Methods**

-Are the objectives of the study clearly articulated with a clear testable hypothesis stated?

-Is the study design appropriate to address the stated objectives?

-Is the population clearly described and appropriate for the hypothesis being tested?

-Is the sample size sufficient to ensure adequate power to address the hypothesis being tested?

-Were correct statistical analysis used to support conclusions?

-Are there concerns about ethical or regulatory requirements being met?

Reviewer #1: Are the objectives of the study clearly articulated with a clear testable hypothesis stated?

Yes. The objectives are clearly articulated and the study addresses a well-defined and testable research question.

Is the study design appropriate to address the stated objectives?

Yes. The study design is appropriate and adequately aligned with the stated objectives.

Is the population clearly described and appropriate for the hypothesis being tested?

Yes. The study population is clearly described and appropriate for the research question.

Is the sample size sufficient to ensure adequate power to address the hypothesis being tested?

Yes. The sample size appears adequate to support the analyses and the study objectives.

Were correct statistical analyses used to support conclusions?

Yes. The statistical analyses are appropriate and support the conclusions drawn.

Are there concerns about ethical or regulatory requirements being met?

No. There are no major concerns regarding ethical or regulatory requirements based on the information provided.

Reviewer #2: The method was clear,well prepared and detailed however minor revision required, please add and clearly state the study population, setting and the sampling in their specific appropriate sections.

Reviewer #3: -Clarify recruitment strategy and representativeness

- Explicit statement of: Ethics approval (with reference number), Informed consent

-The sample size used for psychometric validation is relatively small, especially for analyses such as CFA and subgroup comparisons. While acceptable for an initial validation study, this limitation should be more explicitly acknowledged, and conclusions should be framed accordingly to avoid overgeneralization.

**********

**Results**

-Does the analysis presented match the analysis plan?

-Are the results clearly and completely presented?

-Are the figures (Tables, Images) of sufficient quality for clarity?

Reviewer #1: Does the analysis presented match the analysis plan?

Yes. The analyses presented are consistent with the study aims and appear to match the analysis plan.

Are the results clearly and completely presented?

Yes. The results are clearly organized and sufficiently presented.

Are the figures (Tables, Images) of sufficient quality for clarity?

Yes. The tables and figures are clear, appropriate, and of sufficient quality to support the manuscript.

Reviewer #2: The results were organized and reported the findings of the study objectives, however no items were cited or mentioned and no scale attached.

Reviewer #3: - Currently, it is dense with numerical detail, which may hinder readability. Streamlining the presentation and moving highly detailed outputs to supplementary files would improve accessibility.

**********

**Conclusions**

-Are the conclusions supported by the data presented?

-Are the limitations of analysis clearly described?

-Do the authors discuss how these data can be helpful to advance our understanding of the topic under study?

-Is public health relevance addressed?

Reviewer #1: Are the conclusions supported by the data presented?

Yes. The conclusions are supported by the data presented and remain appropriately balanced.

Are the limitations of analysis clearly described?

Yes. The main limitations are acknowledged and discussed adequately.

Do the authors discuss how these data can be helpful to advance our understanding of the topic under study?

Yes. The manuscript clearly explains how these findings contribute to advancing knowledge in the field.

Is public health relevance addressed?

Yes. The public health relevance is clearly addressed and represents one of the strengths of the manuscript.

Reviewer #2: The conclusion supported with the data but it require revision and clarity and it should be separated from the discussion and kindly organized it with future interventions which help advance the topic and the outcomes of this study.

Please provide a section for the strength and limitations of this study as were not stated in this version of manuscript.

Reviewer #3: - the limitations section should be expanded to include not only sample size constraints but also potential selection bias, limitations inherent to KAP surveys, and constraints in psychometric testing (e.g., limited power for DIF analysis).

**********

**Editorial and Data Presentation Modifications?**

Use this section for editorial suggestions as well as relatively minor modifications of existing data that would enhance clarity. If the only modifications needed are minor and/or editorial, you may wish to recommend “Minor Revision” or “Accept”.

Reviewer #1: Minor revision

Reviewer #2: For the title please revise it to be more reflective and interesting, add the population and revise it.

The heading abstract word is missed, and at the conclusion of the abstract stated that,the study aims to assess the knowledge without mentioning the other objectives. Please add the attitude and practice and rephrase the conclusion to be well reflected.

In the justification of the background please add how this study addresses a gap and identifies how it provides an unexisting instrument.

Reviewer #3: (No Response)

**********

**Summary and General Comments**

Use this section to provide overall comments, discuss strengths/weaknesses of the study, novelty, significance, general execution and scholarship. You may also include additional comments for the author, including concerns about dual publication, research ethics, or publication ethics. If requesting major revision, please articulate the new experiments that are needed.

Reviewer #1: This is a timely, relevant, and well-written manuscript addressing an important educational and public health gap. The topic is highly appropriate for the journal, and the authors should be commended for focusing on preparedness regarding neglected tropical diseases in a high-income European context, where these competencies are often underestimated despite changing epidemiological patterns. Overall, I believe the manuscript is suitable for publication after minor revisions, mainly aimed at improving the clarity, conciseness, and interpretative strength. Below my suggestions:

1.The rationale is strong and convincing, but the final paragraph currently repeats the study aim almost verbatim. I suggest removing the duplicated sentence and slightly tightening the transition from the broader global-health framing to the specific Italian educational context. This would improve readability and make the study objective stand out more clearly.

2.Please improve the balance of the Introduction by focusing more directly on the educational gap.

The opening of the Introduction is broad and well contextualized within globalization, migration, climate change, and health inequities, which is valuable. However, the educational dimension of the problem emerges somewhat late. I would recommend bringing forward, a little earlier, the point that the core issue is insufficient preparedness among medical trainees in high-income settings, so that the narrative becomes more immediately aligned with the specific purpose of the paper.

3. The Discussion appropriately argues that preparedness among young physicians may reflect broader health-system readiness. However, at present these two levels are somewhat blended. I suggest separating more clearly what the study directly supports—namely, curriculum assessment and educational gap identification—from the broader downstream implications for surveillance, preparedness, and health-system responsiveness. This would make the interpretation more precise and proportionate to the study findings.

4, Please expand the practical implications of the tool for curriculum development.

The Discussion rightly emphasizes that the instrument may inform educational reform, but this point could be made even more useful by adding a few concrete examples of application. For instance, the authors might briefly mention its potential use for curriculum mapping, pre/post educational interventions, inter-university comparisons, or assessment of targeted teaching modules in tropical medicine or global health. This would increase the practical value of the paper for educators and policy makers.

5.

The final message is compelling, but it could be strengthened by outlining what the next logical step should be after validation of this national tool. For example, the authors could mention future multicenter use across Italian universities, periodic reassessment of preparedness over time, or eventual adaptation of the instrument for broader European contexts. This would give the Discussion a stronger forward-looking perspective and highlight the manuscript’s wider relevance.

Reviewer #2: This study was well structured, sequenced and organized, however some revisions required specifically in the title of this study, the study justification and conclusion.

Reviewer #3: (No Response)

**********

PLOS authors have the option to publish the peer review history of their article (what does this mean?). If published, this will include your full peer review and any attached files.

If you choose “no”, your identity will remain anonymous but your review may still be made public.

**Do you want your identity to be public for this peer review?**  For information about this choice, including consent withdrawal, please see our Privacy Policy.

Reviewer #1: No

Reviewer #2: No

Reviewer #3: No

[NOTE: If reviewer comments were submitted as an attachment file, they will be attached to this email and accessible via the submission site. Please log into your account, locate the manuscript record, and check for the action link "View Attachments". If this link does not appear, there are no attachment files.]

**Figure resubmission:**

While revising your submission, we strongly recommend that you use PLOS’s NAAS tool (https://ngplosjournals.pagemajik.ai/artanalysis) to test your figure files. NAAS can convert your figure files to the TIFF file type and meet basic requirements (such as print size, resolution), or provide you with a report on issues that do not meet our requirements and that NAAS cannot fix.

After uploading your figures to PLOS’s NAAS tool - https://ngplosjournals.pagemajik.ai/artanalysis, NAAS will process the files provided and display the results in the "Uploaded Files" section of the page as the processing is complete. If the uploaded figures meet our requirements (or NAAS is able to fix the files to meet our requirements), the figure will be marked as "fixed" above. If NAAS is unable to fix the files, a red "failed" label will appear above. When NAAS has confirmed that the figure files meet our requirements, please download the file via the download option, and include these NAAS processed figure files when submitting your revised manuscript.

**Reproducibility:**

To enhance the reproducibility of your results, we recommend that authors of applicable studies deposit laboratory protocols in protocols.io, where a protocol can be assigned its own identifier (DOI) such that it can be cited independently in the future. Additionally, PLOS ONE offers an option to publish peer-reviewed clinical study protocols. Read more information on sharing protocols at https://plos.org/protocols?utm_medium=editorial-email&utm_source=authorletters&utm_campaign=protocols

# Author comment: Author response to Decision Letter 1

**DOI:** 10.1371/journal.pntd.0014644.r002

21 Jun 2026

Attachment Submitted filename: Responses to the Editor and Reviewers.docx

# Peer review: Decision Letter 1

**DOI:** 10.1371/journal.pntd.0014644.r003

**Authors:** Sitara Swarna Rao Ajjampur, Damiano Pizzol

4 Aug 2026

Dear Dr GUIDO,

We are pleased to inform you that your manuscript 'Development and Validation of a National Questionnaire Assessing Knowledge, Attitudes, and Practices on Neglected Tropical Diseases Among Italian Medical Trainee' has been provisionally accepted for publication in PLOS Neglected Tropical Diseases.

Before your manuscript can be formally accepted you will need to complete some formatting changes, which you will receive in a follow up email. A member of our team will be in touch with a set of requests.

Please note that your manuscript will not be scheduled for publication until you have made the required changes, so a swift response is appreciated.

IMPORTANT: The editorial review process is now complete. PLOS will only permit corrections to spelling, formatting or significant scientific errors from this point onwards. Requests for major changes, or any which affect the scientific understanding of your work, will cause delays to the publication date of your manuscript.

Should you, your institution's press office or the journal office choose to press release your paper, you will automatically be opted out of early publication. We ask that you notify us now if you or your institution is planning to press release the article. All press must be co-ordinated with PLOS.

Thank you again for supporting Open Access publishing; we are looking forward to publishing your work in PLOS Neglected Tropical Diseases.

Best regards,

Damiano Pizzol

Guest Editor

PLOS Neglected Tropical Diseases

Sitara Ajjampur

Section Editor

PLOS Neglected Tropical Diseases

Shaden Kamhawi

co-Editor-in-Chief

PLOS Neglected Tropical Diseases

orcid.org/0000-0003-4304-636XX

Paul Brindley

co-Editor-in-Chief

PLOS Neglected Tropical Diseases

orcid.org/0000-0003-1765-0002

***********************************************************

Reviewer's Responses to Questions

**Key Review Criteria Required for Acceptance?**

As you describe the new analyses required for acceptance, please consider the following:

**Methods**

-Are the objectives of the study clearly articulated with a clear testable hypothesis stated?

-Is the study design appropriate to address the stated objectives?

-Is the population clearly described and appropriate for the hypothesis being tested?

-Is the sample size sufficient to ensure adequate power to address the hypothesis being tested?

-Were correct statistical analysis used to support conclusions?

-Are there concerns about ethical or regulatory requirements being met?

Reviewer #1: excellent

Reviewer #3: (No Response)

**********

**Results**

-Does the analysis presented match the analysis plan?

-Are the results clearly and completely presented?

-Are the figures (Tables, Images) of sufficient quality for clarity?

Reviewer #1: NA

Reviewer #3: (No Response)

**********

**Conclusions**

-Are the conclusions supported by the data presented?

-Are the limitations of analysis clearly described?

-Do the authors discuss how these data can be helpful to advance our understanding of the topic under study?

-Is public health relevance addressed?

Reviewer #1: excellent

Reviewer #3: (No Response)

**********

**Editorial and Data Presentation Modifications?**

Use this section for editorial suggestions as well as relatively minor modifications of existing data that would enhance clarity. If the only modifications needed are minor and/or editorial, you may wish to recommend “Minor Revision” or “Accept”.

Reviewer #1: NA

Reviewer #3: (No Response)

**********

**Summary and General Comments**

Use this section to provide overall comments, discuss strengths/weaknesses of the study, novelty, significance, general execution and scholarship. You may also include additional comments for the author, including concerns about dual publication, research ethics, or publication ethics. If requesting major revision, please articulate the new experiments that are needed.

Reviewer #1: The authors have written an important paper with great methodological rigour. From my point of view, this is an excellent manuscript.

Reviewer #3: The authors have satisfactorily addressed all reviewer comments and have made appropriate revisions throughout the manuscript. The changes have significantly improved the quality, transparency, and readability of the work. In my opinion, the manuscript is now suitable for publication, and I have no further revisions to request.

**********

PLOS authors have the option to publish the peer review history of their article (what does this mean?). If published, this will include your full peer review and any attached files.

If you choose “no”, your identity will remain anonymous but your review may still be made public.

**Do you want your identity to be public for this peer review?**  For information about this choice, including consent withdrawal, please see our Privacy Policy.

Reviewer #1: No

Reviewer #3: No

# Editor report: Acceptance letter

**DOI:** 10.1371/journal.pntd.0014644.r004

**Authors:** Sitara Swarna Rao Ajjampur, Damiano Pizzol

Dear Dr GUIDO,

We are delighted to inform you that your manuscript, "Development and Validation of a National Questionnaire Assessing Knowledge, Attitudes, and Practices on Neglected Tropical Diseases Among Italian Medical Trainee," has been formally accepted for publication in PLOS Neglected Tropical Diseases.

We have now passed your article onto the PLOS Production Department who will complete the rest of the publication process. All authors will receive a confirmation email upon publication.

The corresponding author will soon be receiving a typeset proof for review, to ensure errors have not been introduced during production. Please review the PDF proof of your manuscript carefully, as this is the last chance to correct any scientific or type-setting errors. Please note that major changes, or those which affect the scientific understanding of the work, will likely cause delays to the publication date of your manuscript. Note: Proofs for Front Matter articles (Editorial, Viewpoint, Symposium, Review, etc...) are generated on a different schedule and may not be made available as quickly.

Soon after your final files are uploaded, the early version of your manuscript will be published online unless you opted out of this process. The date of the early version will be your article's publication date. The final article will be published to the same URL, and all versions of the paper will be accessible to readers.

For Research Articles, you will receive an invoice from PLOS for your publication fee after your manuscript has reached the completed accept phase. If you receive an email requesting payment before acceptance or for any other service, this may be a phishing scheme. Learn how to identify phishing emails and protect your accounts at https://explore.plos.org/phishing.

Thank you again for supporting open-access publishing; we are looking forward to publishing your work in PLOS Neglected Tropical Diseases.

Best regards,

Shaden Kamhawi

co-Editor-in-Chief

PLOS Neglected Tropical Diseases

Paul Brindley

co-Editor-in-Chief

PLOS Neglected Tropical Diseases
